# Comparative analysis of allele frequencies for DNA polymorphisms associated with disease and economically important traits in the genomes of Russian and foreign cattle breeds

**DOI:** 10.18699/VJGB-22-28

**Published:** 2022-05

**Authors:** A.V. Igoshin, G.A. Romashov, E.N. Chernyaeva, N.P. Elatkin, N.S. Yudin, D.M. Larkin

**Affiliations:** Institute of Cytology and Genetics of the Siberian Branch of the Russian Academy of Sciences, Novosibirsk, Russia; Institute of Cytology and Genetics of the Siberian Branch of the Russian Academy of Sciences, Novosibirsk, Russia; LLC “Miratorg-Genetika”, Moscow, Russia; LLC “Miratorg-Genetika”, Moscow, Russia; Institute of Cytology and Genetics of the Siberian Branch of the Russian Academy of Sciences, Novosibirsk, Russia; Institute of Cytology and Genetics of the Siberian Branch of the Russian Academy of Sciences, Novosibirsk, Russia Royal Veterinary College, London, United Kingdom

**Keywords:** cattle, selection, breed, Russian Federation, genetic variants, SNP, insertion, deletion, крупный рогатый скот, селекция, порода, Российская Федерация, генетические варианты, SNP, инсерция, делеция

## Abstract

The genetic makeup of a breed including its genetic differences from other breeds determines its appearance and characteristics, including economically important traits and resistance to pathologies. To date, many loci controlling significant phenotypes have been identified, which is successfully used in the world practice of marker-assisted selection to improve breed properties. The aim of this study was a comparative analysis of frequencies for known causative nucleotide substitutions, insertions and deletions associated with disease and economically important traits in Russian and foreign cattle breeds. As a result, we identified frequencies of these DNA polymorphisms in the populations of Russian cattle breeds, compared them with those of foreign populations of the same breed, as well as other foreign breeds. Our results indicate similarities in frequencies for most of such alleles within breeds (populations of Russian and foreign breeding), as well as the relationship between the causative allele prevalence and the presence of phenotypic traits under the effect. We also found an excess of some undesirable alleles in the Russian cattle populations, which should be paid attention to when designing breeding programs. We found that the alleles increasing fertility in the Hereford breed have a higher frequency in the Russian Hereford population compared to the foreign counterpart. Interestingly, unlike for the European breeds, for Asian Turano-Mongolian Wagyu and Yakut cattle, there was a less clear link between phenotypic traits and frequencies of known causative alleles. Our work points to specific genetic variants that could be used to improve and/or maintain the performance of certain cattle breeds bred in the Russian Federation

## Abstract (ru)

Генетический состав породы и ее генетические отличия от других пород определяют ее облик и характерные особенности, включая экономически важные признаки и встречаемость патологий. К настоящему времени выявлено множество локусов, контролирующих наиболее значимые фенотипы, что успешно используется в мировой практике для маркер-ассоциированной селекции в целях улучшения свойств пород. В настоящей работе проведен сравнительный анализ частот известных каузативных нуклеотидных замен, вставок и делеций, связанных с заболеваниями и хозяйственно ценными признаками, в российских и зарубежных породах крупного рогатого скота. Выявлены частоты вышеуказанных ДНК-полиморфизмов в популяциях российских пород крупного рогатого скота, выполнено их сравнение с частотами в зарубежных популяциях для пород, разводимых в Российской Федерации, а также с другими зарубежными породами. Наши результаты показывают схожесть частот большинства аллелей внутри пород (российского или зарубежного разведения), а также связь между представленностью аллелей исследуемых полиморфизмов и наличием определяемых ими фенотипических признаков. Были найдены и превышения по частотам ряда нежелательных аллелей в российских популяциях крупного рогатого скота, на которые стоит обратить внимание при селекционной работе с породами. Обнаружено, что аллели, отвечающие за повышенную фертильность породы герефорд, имеют повышенную частоту в популяциях российского разведения по сравнению с зарубежными популяциями. Интересно, что для азиатских турано-монгольских вагю и якутского скота наблюдалась меньшая связь между фенотипическими признаками и частотами известных каузативных аллелей по сравнению с европейскими породами. Наша работа указывает на конкретные генетические варианты, которые могут быть использованы для улучшения и/или поддержания качеств ряда пород крупного рогатого скота, разводимых в Российской Федерации

## Introduction

Common types of genetic variations, such as single nucleotide polymorphisms, nucleotide insertions and deletions, among others, can have “beneficial” or “harmful” effects on animal health and productivity (Liu, Bickhart, 2012; Bourque et al., 2018). That is why the sequencing of the Bos taurus genome caused a surge in research on the genetic diversity of cattle breeds and its relationship with economically important traits, adaptations and diseases, which opened up opportunities to use the knowledge gained for creating breeds with the necessary qualities and improving existing breeds (Larkin, Yudin, 2016; Yudin, Larkin, 2019). Now, according to the OMIA database (www.omia.org; Lenffer et al., 2006), 272 bovine traits are known to be genetically controlled, including a number of diseases. For 175 of them, causative mutations in the coding and non-coding regions of DNA have already been identified, the effect of which is related to various mechanisms, including changes in the protein sequence, in the stability, expression or processing of RNA (Ibeagha-Awemu et al., 2008; Yudin, Voevoda, 2015; Ciepłoch et al., 2017). Using this information, tests were developed for genotyping pathological mutations and removing carrier animals from the breeding herd (Romanenkova et al., 2015; Fornara et al., 2019; Sabetova et al., 2021). With this approach, it is possible to identify mutations at an early age for the timely culling of animals or embryos (Terletskiy et al., 2016). At the same time, it is worth considering that a “harmful” mutation may be “useful” for another economically important trait (Fasquelle et al., 2009). Identification of gene alleles associated with economically important traits allowed using them for marker-assisted selection (Pighetti, Elliott, 2011; Abd El-Hack et al., 2018). Marker-assisted selection is particularly important for traits that become evident with age or only in animals of the same sex, such as productivity or fertility (Zinovieva, 2016; Raina et al., 2020).

So far, Russian cattle breeds have been investigated for the presence of only a few, the most common mutations associated with economically important traits and health (Romanenkova et al., 2016, 2018; Usova et al., 2017; Surzhikova et al., 2019). The purpose of our work was to analyze the spectrum and frequencies of known causative DNA polymorphisms in nine Russian cattle breeds using genome sequencing data and to compare the frequencies of these polymorphisms with those in worldwide breeds or foreign populations of the same breeds to determine the options for which the selection in Russian cattle could be conducted.

## Materials and methods

The list of single-nucleotide polymorphisms (SNPs), insertions and deletions, clinically and economically important for cattle, was compiled based on the information from the OMIA database (www.omia.org; Lenffer et al., 2006) and practical guidance of the Irish Cattle Breeding Federation (McClure M., McClure J., 2016). The genomic positions of polymorphisms specified in the Bos taurus UMD3.1 assembly coordinates were converted to the ARS-UCD1.2 assembly coordinates using liftOver (Kuhn et al., 2013). For polymorphisms present in the sample of Russian breeds, reference and alternative alleles were verified for matching those specified in the publications. For four substitutions of the twelve possible (T↔A and G↔C), such a verification is complicated, since: (1) there may be a change in the reference allele during the transition to a new genome assembly; (2) in the publication, the allele can be specified for a chain which is complementary to reference sequence. In such cases, we verified the alleles of polymorphisms in the context of codons (for substitutions in the coding sequence) or proximate sequences. For example, according to Hirano and colleagues (2013) and the OMIA database, replacing the nucleotide G with C at the BTA8:83909754 position, leading to the replacement of valine with leucine, results in perinatal weak calf syndrome. However, apparently, this replacement was indicated by the authors for the messenger RNA sequence – since in the assembly ARS-UCD1.2 C stands for the reference nucleotide, being a part of the “AAC” triplet, which, in turn, corresponds to “GUU” mRNA codon, encoding valine. Thus, in the reference assembly of ARS-UCD1.2, the G allele will be “harmful”.

In this paper, we used data on SNPs, insertions and deletions in the worldwide breeds from the “1000 Bull Genomes” Project (Hayes, Daetwyler, 2019), including the resequencing data of eight Russian breeds obtained earlier, as well as the resequencing data (“.fastq”-files) for the Russian population of the Aberdeen Angus breed (hereinafter simply Angus), provided by LLC “Miratorg-Genetika”. Of note, some of these animals were imported from the USA and Australia (Table 1). Additionally, we also used data on three native Finnish breeds provided by the Natural Resources Institute Finland (Luke). Finland borders with Russia and has a largely similar (although milder) climate, so the inclusion of Finnish breeds in the study could shed light on features of the selective breeding manifesting in the close natural conditions of the two countries.

**Table 1.**
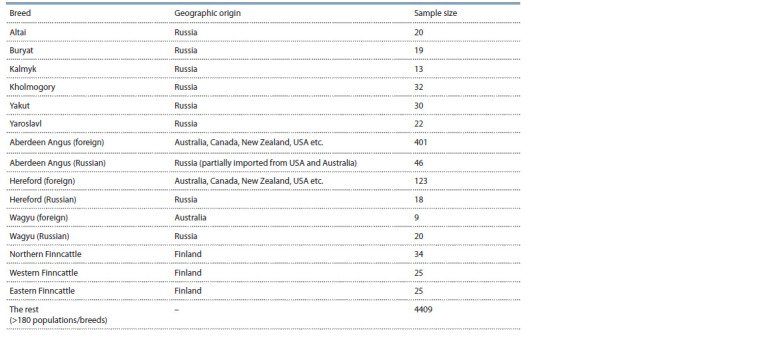
Breed analyzed

Removal of adapter sequences from raw paired reads was performed using Trimmomatic-0.39. Clean reads were aligned to the ARS-UCD1.2 reference sequence using BWA-MEM v.0.7.17 (Li, Durbin, 2009). Files containing aligned sequences (“.sam”-files) were then converted to the “.bam” format and sorted using the SAMtools v.1.8 software (Li et al., 2009). Further, libraries belonging to the same animal were pooled using the ‘MergeSamFiles’ module of the Picard v.2.18.2 package (http://broadinstitute.github.io/ picard). Duplicates were marked using the ‘MarkDuplicates’ module of the above-mentioned software. The OPTICAL_ DUPLICATE_PIXEL_DISTANCE parameter equaling 2500 was chosen according to the recommendations of the “1000 Bull Genomes” protocol. Base quality score recalibration was performed using the ‘BaseRecalibrator’ and ‘PrintReads’ modules of the GATK v.3.8 package (McKenna et al., 2010) using data provided by the “1000 Bull Genomes” Project (Hayes, Daetwyler, 2019). The variant calling and the merging of the resulting gVCF files were performed using the ‘HaplotypeCaller’ and ‘GenotypeGVCFs’ modules of the GATK v.3.8 program, respectively.

Extraction of SNPs, insertions, and deletions from genomewide VCF files was performed with the Tabix utility (Li, 2011), using the coordinates of polymorphisms from a previously generated list. The resulting VCF files containing the selected polymorphisms were used to calculate the frequencies of alternative alleles in the samples using the PLINK 2.0 program (Purcell et al., 2007) with the following parameters: --vcf --chrset 30 --freq --pheno --loop-cats. The count has been carried out for (1) breeds bred in Russia (Kholmogory, Yaroslavl, Altai, Yakut, Buryat, Kalmyk, Angus, Wagyu and Hereford), (2) foreign populations of those breeds (if present), (3) three Finnish breeds (Northern Finncattle, Western Finncattle and Eastern Finncattle), and (4) a combined sample of all other worldwide cattle breeds (see Table 1).

The presence of allele frequency differences between the abovementioned samples was tested using Fisher’s exact test implemented in the ‘fisher.test()’ R function. Contingency tables 2×2 were composed by counting the number of reference and alternative alleles in the chromosomal pool of each of the two groups studied. Three types of comparisons were made: (1) between a breed bred in Russia (or a foreign population of the same breed, if present) and a combined sample of other world breeds; (2) between a breed bred in Finland and the combined sample of the world’s breeds using the polymorphisms identified in the first type of comparisons; (3) only between the Russian population and the foreign population of the same breed. To correct for multiple comparisons, we used the Storey and Tibshirani method (Storey, Tibshirani, 2003) implemented in the ‘qvalue()’ R function (Storey et al., 2020).

## Results

Our list of clinically and economically important polymorphisms contained 193 SNPs and 63 insertions/ deletions. A search in the VCF files revealed in Russian breeds the presence of 38 SNPs and one insertion from the abovementioned list (Supplementary Table 1)1, which corresponded to at least 21 phenotypic traits

Supplementary Tables 1 and 2 are available in the online version of the paper: http://vavilov.elpub.ru/jour/manager/files/Suppl_Igoshin_engl.pdf


When comparing 15 populations for 39 polymorphisms (585 comparisons in total) with a global sample, in 229 cases statistically significant (q < 0.05) differences in allele frequencies were found (see the Figure). The most significant differences with the total sample of worldwide breeds were observed for foreign populations of Angus and Hereford breeds (29 and 27 loci, respectively). Of the Russian populations, the Yakut breed had the largest number (16 loci) of differences from the worldwide sample. Of the Finnish breeds, the Northern Finncattle had the largest number (20 loci) of such differences

**Fig. 1.**
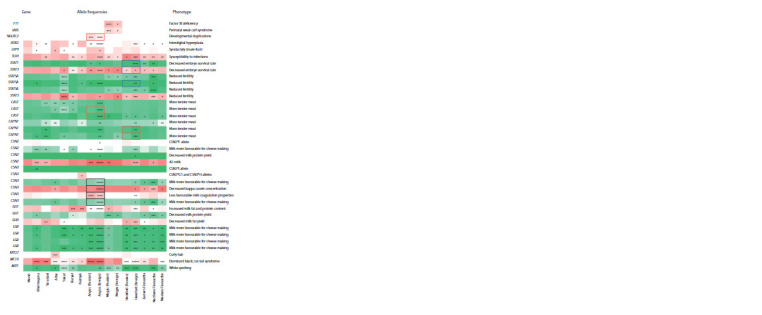
Frequencies of clinically and economically significant polymorphisms in Russian and foreign cattle populations

On the left are the genes containing the polymorphic variants under study. A darker tone corresponds to a higher frequency of a reference (green) or alternative (red) allele. On the right is the phenotype associated with this allele. The polymorphism designations and their frequencies are given in Suppl. Table 2. The asterisks indicate loci that have significant frequency differences between the specified breed and the global cattle population: ***** q <1.0E–25, **** q < 1.0E–10, *** q < 1.0E–5, ** q < 1.0E–3, * q < 0.05. The frame indicates loci that differ (p <0.05) between the Russian and foreign populations of the same breed: red color means that the “harmful” allele has a large content in the Russian population, blue – in the foreign, black – the significance of the allele for beef breeds is not established.

The most significant (q = 4.24E–286) allele frequency difference from the global sample was observed for the foreign Angus population for SNP rs109688013 in the melanocortin-1 receptor gene MC1R, carriers of the alternative allele C of which have a black coat color (Klungland et al., 1995). The difference from the worldwide sample for this locus was also statistically significant for most of other populations as well, with the exception of the Northern Finncattle, as well as Russian and foreign Wagyu populations. In particular, the difference at this SNP was the highest among 39 loci for the Russian population of Angus (q = 6.01E–35), both populations of Herefords (q = 6.22E–37 for foreign and 7.34E–07 for Russian), for Altai (q = 1.99E–06), Kholmogory (q = 9.27E–12) and Yaroslavl (q = 2.76E–06) breeds. In foreign and Russian Angus populations, the frequency of the C allele coding for black color reaches 0.973 and 0.989, while in other worldwide breeds it is 0.339. In the populations of Altai, Kholmogory, Yaroslavl breeds, Russian and foreign Herefords, it has a frequency of 0.026, 0.828, 0.772, 0 and 0.019, respectively. In Finnish breeds, the frequency of the C allele varies from zero in Western Finncattle to 0.052 in Eastern Finncattle and 0.258 in Northern Finncattle.

Of the remaining loci, the greatest difference in the studied breeds from the global cattle population was observed for polymorphisms associated with milk traits, coat color and bleeding disorders. Thus, the Russian Wagyu population had the most significant (q = 6.44E–21) allele frequency difference from the worldwide sample for 15 bp insertion located at BTA27:16305660, which disrupts the F11 gene function and, as a result, leads to a deficiency of blood coagulation factor XI, encoded by this gene (Kunieda et al., 2005). In Russian Wagyu population, the frequency of this insertion reaches 0.25, while in the global cattle population it is close to zero. The most significant differences from the worldwide sample for the foreign Wagyu population (q = 2.60E–05) and the Yakut breed (q = 2.21E–18) were observed for SNP rs210634530 in the gene of microphthalmia-associated transcription factor MITF, which is associated with the ‘white spotting’ phenotype (Fontanesi et al., 2012). The frequencies of the ‘white spotting’-associated allele T in the Yakut breed and foreign Wagyu population are 0.083 and 0.111, respectively, while in the worldwide sample it reaches 0.65. In the Buryat and Kalmyk breeds, the most significant difference (q = 6.81E–10 and 2.33E–06, respectively) had SNP rs109191047 in the growth hormone gene GH1, associated with the composition of milk (Mullen et al., 2010). The frequency of G allele increasing the milk fat and protein content is 0.100 in the worldwide population, while in the above-mentioned breeds it reaches 0.526 and 0.500, respectively

Comparisons between the Russian and corresponding foreign populations, made for the Angus, Hereford and Wagyu breeds, revealed four loci, statistically significantly (q < 0.05) differing in allele frequencies. Of these, three SNPs (rs43703017, rs43703015 and rs110014544) had differing frequencies in the Russian and foreign Angus populations and specified the alleles of the kappa-casein gene CSN3. One SNP located in the CAPN1 gene (rs17872050) differed between the Hereford populations and was associated with meat tenderness. Taking into account the frequency differences at the nominal significance level (p < 0.05), eight additional loci can be noted (Table 2), among which the V311A missense substitution (BTA26:34340886T>C) in the NHLRC2 gene differing between the Angus populations and in homozygotes leading to notomelia, a type of polymelia in which the additional limb is located along or near the midline of the back (Beever et al., 2014).

**Table 2.**
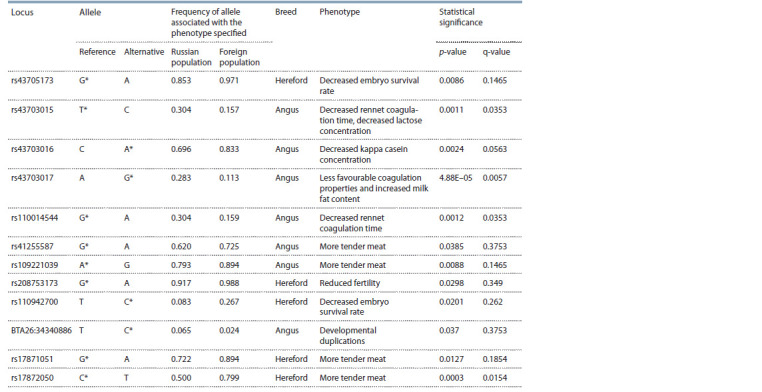
Differences between Russian and foreign populations within the same breed The allele associated with the phenotype specified

## Discussion

Breed-specific genetic features

The gene pool of farm animals is formed under the influence of factors such as selection for productive traits, adaptation to environmental conditions, hybridization, de novo mutations, the founder effect and genetic drift (Notter, 1999; Xu et al., 2015).

As we showed above, a significant part of the polymorphisms taken into the analysis in the studied breeds differs in frequencies from the “worldwide average”, reflecting the gene pool features of particular populations. For example, the Yakut cattle shows the highest divergence in allele frequencies among Russian breeds, expressed both in a greater number of differing loci and in a greater significance of these differences, which is consistent with the data on phylogeny of this breed and the analysis of its population structure (Yurchenko et al., 2018; Buggiotti et al., 2021).

Some of the polymorphisms studied make a definitive contribution to characteristic features of the breeds. For example, the content of the MC1R gene allele rs109688013-C in the breeds coincides well with the typical color of their representatives. Thus, in Angus having a black coat color, the frequency of this allele is close to one. In Yaroslavl and Kholmogory cattle, rs109688013-C also predominates, apparently defining black and black-mottled coats, mainly characteristic of these animals. At the same time, in Herefords, which are not characterized by a black color, the frequency of the C allele is close to zero. Similarly, there is a link between color and the frequency of the C allele in populations of Finnish breeds. In breeds that have mainly lighter coats (fawn, light brown and red, often white muzzle, belly and back), it is low (0.053 in Eastern Finncattle) or zero (in Western Finncattle). In Northern Finncattle, which has a predominantly white coat color (some individuals are black-mottled), the frequency of rs109688013-C is 0.258. Breeds for which the red (Kalmyk) or brown (Altai, Buryat) colors are typical have rs109688013-C in low frequency (0.03–0.08). However, in Wagyu populations, which are usually characterized by black color, the frequency of this allele is far from one and has values of 0.42 in Russian population and 0.67 in foreign population, probably reflecting the genetic characteristics of Turano- Mongolian breeds. This discrepancy is also observed in Yakut cattle, in which a black-and-white color is common, but the frequency of rs109688013-C is vanishingly small. Given the genetic divergence of Turano-Mongolian breeds from other breeds, it can be assumed that other loci are involved in the control of body coloration.

Also, coat color is associated with the SNP rs210634530 in the MITF gene, the T allele of which defines the ‘white spotting’ phenotype. The highest frequency of rs210634530-T is observed in populations of Hereford cattle (fixed in the Russian sample and 0.92 in the foreign population), which is characterized by a white head and belly. In addition, this allele prevails in the populations of Kholmogory, Yaroslavl, Altai and Kalmyk breeds, which have white spotting in color, as well as in Angus. In other populations, the frequency of the T allele varies from low (Yakut breed) to moderate (Buryat, Wagyu). The link between the content of rs210634530-T and coat color can be demonstrated for Finnish breeds. As mentioned above, many individuals of Western Finncattle and Eastern Finncattle have a white muzzle, back, and belly. Northern Finncattle has either a white or, less often, black-and-white coat. It should be noted that in addition to SNP rs210634530, additional loci appear to be involved in the control of the ‘white spottingʼ phenotype (Fontanesi et al., 2012), therefore, the link between the frequency of rs210634530-T and coat color may not be so straightforward.

Some of the genetic features of the breeds are not quite obvious at first glance. For example, both Russian and foreign populations of Angus and Wagyu have a high (0.89–0.95) frequency of rs43703011-G allele of the beta-casein gene CSN2. Variations of the CSN2 gene at several non-synonymous positions determine its alleles – A1, A2, A3, B, C, etc. The above-mentioned allele G of rs43703011 is shared by several alleles of the CNS2 gene, the most common of which is A2. The so-called A2-milk is considered more preferable for consumption, due to better absorption and fewer undesirable effects from the human digestive system (Jianqin et al., 2016). In recent years, breeding programs in many countries have aimed to increase the frequency of the A2 allele in dairy cattle (Sebastiani et al., 2020). Given that Angus and Wagyu are beef breeds and are not used for milk production, the increased G allele content they have can hardly be explained by selection to improve milk quality. The most plausible explanation is selection for meat productivity. Thus, according to Hohmann et al. (2020), the carriage of the A2 allele increases average daily weight gain and weaning weight in German Angus and Simmentals. Therefore, increasing the frequency of the rs43703011-G allele, and consequently the A2 allele of the CSN2 gene, can be useful for improving not only dairy but also beef breeds.

Some of the variants found are specific to one breed and virtually absent in others. The most breed-specific are clinically important polymorphisms in the F11, IARS and NHLRC2 genes. The previously mentioned insertion in the F11 gene, leading to a deficiency of blood coagulation factor XI, is almost exclusively observed in foreign and Russian Wagyu populations. At the same time, among more than 5 thousand other animals from the “1000 Bull Genomes” Project, this mutation is harbored by only two animals. Association of factor XI activity with ATATGTGCAGAATAT insertion has been initially demonstrated for Wagyu (Kunieda et al., 2005). The homozygous genotype for this mutation is associated with a blood clotting disorder and an increase in the duration of bleeding. In the Russian population of Wagyu, its frequency equals 0.25, which is consistent with the data of early publications on its prevalence in the Japanese black breed (Watanabe et al., 2006; Ohba et al., 2008). At the same time, in the foreign Wagyu population, here represented by a sample from Australia, this insertion has a frequency of 0.11.

Other examples of breed-specific variants are single nucleotide substitutions in the IARS (BTA8:83909754C>G) and NHLRC2 (BTA26:34340886T>C) genes. BTA8:83909754C>G mutation in the IARS gene in homozygote leads to perinatal weak calf syndrome and increased prenatal mortality (Hirano et al., 2013, 2016). This variant is specific to Wagyu, and besides, it was found only in one animal from the “1000 Bull Genomes” Project. In the Russian and Australian samples of this breed, its frequencies are 0.075 and 0.056, respectively. The BTA26:34340886T>C mutation in the NHLRC2 gene mentioned earlier, in homozygote leading to notomelia, is breed-specific for Angus, and was first discovered in this breed (Beever et al., 2014). Besides Angus, in the sample of “1000 Bull Genomes”, the mutant allele is found only in one animal of an unknown (‘crossbreed’) breed. In the Russian and foreign populations of this breed, it has frequencies of 0.065 and 0.024, respectively.

Differences between Russian and foreign populations of the same breed

The presence in our analysis of foreign Angus, Herefords and Wagyu populations can shed light on the features of the selection and adaptation of Russian populations of these breeds. Overall, Russian and foreign samples of the same breed demonstrate similar allele frequency profiles, with statistically confirmed differences present only in a small number of loci. The differences observed can be explained by many factors or their combinations. For example, an almost threefold excess of the BTA26:34340886-C allele (leading to the appearance of additional limbs) content in the Russian Angus population compared to the foreign one (see Table 2) may be a consequence of the founder effect or genetic drift in general, as well as less intensive efforts for elimination of this variant in the Russian herd.

Interpopulation differences in the loci associated with reproduction may result from an adaptation to environmental conditions. In the Russian population of Herefords, alleles of several polymorphisms that negatively affect the survival of embryos (rs43705173-G and rs110942700-C) and fertility (rs208753173-G) have a lower frequency compared to the foreign sample of this breed. It can be assumed that the Russian sample of Herefords, in this work represented by a population bred in Western Siberia since the 1960s (Vsyakikh, Kurinsky, 1976), was subject to selection for reproductive performance. This assumption is supported by the data of Afanasyeva and co-authors, according to which in the conditions of the Altai Territory, the population of Herefords of Siberian selection shows a much lower stillbirth rate (1.4 %) compared to animals of Finnish selection (6.6 %) imported in 2011 (Afanasyeva et al., 2015). Low temperatures are known to negatively affect the reproduction of cattle, reducing fertility and increasing perinatal mortality (Gwazdauskas, 1985; Mee, 2020). Therefore, population differences at these loci may reflect the process of genetic adaptation aimed at compensating a decrease in reproductive functions caused by cold.

Of particular interest are single nucleotide polymorphisms associated with meat traits and differing in samples of Angus (rs41255587 and rs109221039 in the CAST gene) and Herefords (rs17871051 and rs17872050 in the CAPN1 gene). For all four SNPs, the foreign populations of these breeds demonstrate a higher content of alleles increasing meat tenderness. This is an important gastronomic feature and its improvement is included in the breeding programs of foreign beef breeds (Tatum, 2006). At the same time, we are not aware of extensive breeding attempts of this kind in Russia, which is probably the reason for the observed differences between the samples. Therefore, the Russian populations of Angus and Herefords have the potential for the improvement of meat quality by selection for CAST and CAPN1 alleles.

Of the studied loci differing between populations of the same breed, four SNPs (rs43703015, rs43703016, rs43703017 and rs110014544) determining the kappa-casein gene CSN3 alleles deserve to be noticed. Their allele frequencies differ between Russian and foreign Angus populations. These polymorphisms are associated with milk traits, in particular, with the concentration of kappa-casein in milk and milk coagulation properties, which is important for cheesemaking. At the same time, the effect of CSN3 alleles on the productivity of beef cattle is poorly understood. Investigations of Tambasco et al. (2003) и Curi et al. (2005) found no association between CSN3 alleles and meat traits. Thus, the observed differences can be attributed to the founder effect, or selection for economically important traits whose associations with CSN3 polymorphisms have not yet been identified

Polymorphisms of clinical significance present in Russian breeds

In Russian breeds, there is a number of polymorphic variants, in homozygous state causing hereditary diseases, some of which (mutations in the genes F11, IARS and NHLRC2) have already been discussed above due to their breed specificity. Also, the variants in the ROR2 and LRP4 genes should be mentioned that are associated with the manifestation of interdigital hyperplasia (proliferation of tissue between the hooves) and syndactyly (fusion of the fingers, also called ‘mule foot’), respectively. Unlike the F11, IARS and NHLRC2 genes, the “harmful alleles” in ROR2 (rs377953295-A) and LRP4 (rs453049317-T) are not breed-specific, and are widespread both in Russian breeds and in the rest of the worldwide cattle population. Of the Russian populations, the Kalmyk (0.192) and Altai (0.15) breeds have the highest content of the rs37795322-A allele of the ROR2 gene. In the worldwide sample, its frequency reaches 0.13. The rs453049317-T variant in the LRP4 gene has the highest frequency in the Altai breed (0.2) and in the Russian Angus (0.12), while in the rest of the worldwide population it is 0.076.

Currently, testing for genetic defects is widely used in the practice of animal husbandry in many countries (Terletskiy et al., 2016). For example, testing for mutations in the F11 and IARS genes is included in the genetic screening programs recommended by the Australian Wagyu Association (https://www.wagyu.org.au/content/uploads/2020/08/ Genetic-Conditions-in-Wagyu-FactSheet-2020.pdf). At the same time, the elimination of undesirable alleles should be approached with caution. For example, there is an assumption that the carriage of mutations associated with syndactyly improves the milk productivity of cows, which can partially explain the spread of this pathology in cattle (Johnson et al., 2006).

## Conclusion

Our analysis showed the allele frequency distribution for the most clinically and economically important DNA polymorphisms present in Russian cattle breeds. A number of variants leading to common hereditary disorders in cattle have significant representation in Russian populations, and probably need to be eliminated. Also, the differences between Russian and foreign cattle populations at several loci are presumably of adaptive importance. The data of this study may be useful in cattle breeding programs aimed at improving the existing cattle breeds, and creating new ones.

## Conflict of interest

The authors declare no conflict of interest.
